# Construction of Chiral Nanoassemblies Based on Host-Guest Complexes and Their Responsive CD and CPL Properties: Chirality Transfer From 2,6-helic[6]arenes to a Stilbazolium Derivative

**DOI:** 10.3389/fchem.2019.00543

**Published:** 2019-08-02

**Authors:** Yan Guo, Ying Han, Chuan-Feng Chen

**Affiliations:** ^1^Beijing National Laboratory for Molecular Sciences, CAS Key Laboratory of Molecular Recognition and Function, Institute of Chemistry, Chinese Academy of Sciences, Beijing, China; ^2^University of Chinese Academy of Sciences, Beijing, China

**Keywords:** helic[6]arene, host-guest complexation, self-assembly, chirality transfer, circularly polarized luminescence

## Abstract

A couple of water-soluble chiral 2,6-helic[6]arene derivatives ***P***-**H1** and ***M***-**H1** were synthesized, and they could form 1:1 stable complexes with 4-[(4′-*N, N*-diphenylamino)-styryl]-*N*-methylpyridinium iodide (**G**) in water. Compared with **G**, the host-guest complexes exhibited enhanced fluorescence, which might be attributed to the spatial confinement of **G** and restriction of aggregation-caused quenching (ACQ) effects. Based on the host-guest complexation, the first helic[6]arene-based chiral assemblies were then constructed, and they showed rectangular or hexagonal nanostructures by scanning electron microscopy (SEM) images. Interestingly, the assemblies showed clear mirror-image circular dichroism (CD) and circularly polarized luminescence (CPL) spectra in aqueous solution, revealing a consecutive chirality transfer from the chiral macrocyclic cavities of the hosts to **G**. Moreover, the supramolecular chirality of the assemblies could also show responsiveness to the pH values and temperatures of the system.

## Introduction

Circularly polarized luminescent (CPL) materials have aroused extensively interest for their potential applications in the fields of biological probes (Carr et al., 2012), photoelectric devices (Grell et al., 2001; Shimada et al., 2017; Li et al., 2018a), asymmetric synthesis (Kawasaki et al., 2005; Xu et al., 2014), and chiral sensing (Yang et al., 2013). It is well-known that chirality and luminophores are two essential factors to realize CPL, and most organic CPL materials combined the two factors, but the construction of the materials is generally inconvenient (Han et al., 2017; Li et al., 2017; Chen et al., 2018). As an alternative way, supramolecular assembling based on the complexation motif between a chiral host and an achiral organic fluorescent dye will be convenient and efficient for construction of the CPL materials (Liu et al., 2015). Recently, Inouye's group reported two doubly threaded [4]rotaxanes with strong CPL based on γ-cyclodextrins (Inouye et al., 2014; Hayashi et al., 2018). Liu's group reported a pyrene-cyclodextrin supra-dendron which showed 1D and 2D nanostructures with CPL activities (Zhang Y. et al., 2018). Because of their commercial availability and chiral cavities, cyclodextrins were often utilized as hosts to construct chiral assemblies or nanostructures based on the chirality transfer motifs (Maeda et al., 2011; Yamaguchi et al., 2011; Sun et al., 2013; Yoshihara et al., 2013; Zhang et al., 2014; Zhang W. et al., 2016; Krishnan and Gopidas, 2017; Zhang B. et al., 2018). However, single enantiomer of cyclodextrins would affect their potential applications in chiral functional materials to some extent. Especially, the lack of chiral macrocyclic hosts could limit the development of such a research area in the host-guest complexation induced CLP materials. As a result, the examples on supramolecular assemblies with CPL properties based on host-guest complexation motif are very limited. Moreover, still no such assemblies with responsive CPL activities have been reported so far.

**Figure 1:**
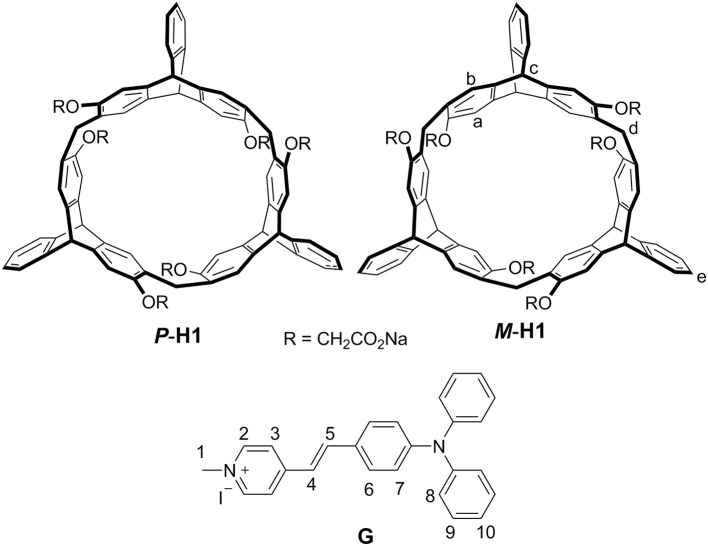
Structures and proton designations of hosts ***P*-H1**/***M***-**H1** and guest **G**.

Recently, we reported a new type of chiral macrocyclic arenes, 2,6-helic[6]arenes (Zhang G. W. et al., 2016; Chen and Han, 2018), which could not only show efficient and enantioselective recognition toward chiral organic ammonium salts, but also form host-guest complexes with various organic guests (Shi and Chen, 2017; Shi et al., 2017; Zhang et al., 2017a,b,c; Wang et al., 2018). It was further deduced that chiral macrocyclic arenes could provide an opportunity to develop the CPL materials based on the host-guest complexation. In this paper, we report a couple of water-soluble chiral 2,6-helic[6]arene derivatives ***P***-**H1** and ***M***-**H1**, which could form 1:1 stable complexes with 4-[(4′-*N, N*-diphenylamino)styryl]-*N*-methylpyridinium iodide (**G**) in water (Figure 1). Compared with **G**, the host-guest complexes exhibited enhanced fluorescence, which might be attributed to the spatial confinement of **G** and restriction of aggregation-caused quenching (ACQ) effects. Based on the host-guest complexation, the first helic[6]arene-based chiral assemblies with rectangular or hexagonal nanostructures were then constructed. Interestingly, the supramolecular assemblies showed clear mirror-image CD and CPL spectra in aqueous solution, revealing a consecutive chirality transfer from the chiral macrocyclic cavities to **G**. Moreover, the assemblies could also show the pH and temperature responsive CD and CPL properties.

## Results and Discussion

### Synthesis

**Scheme 1:**
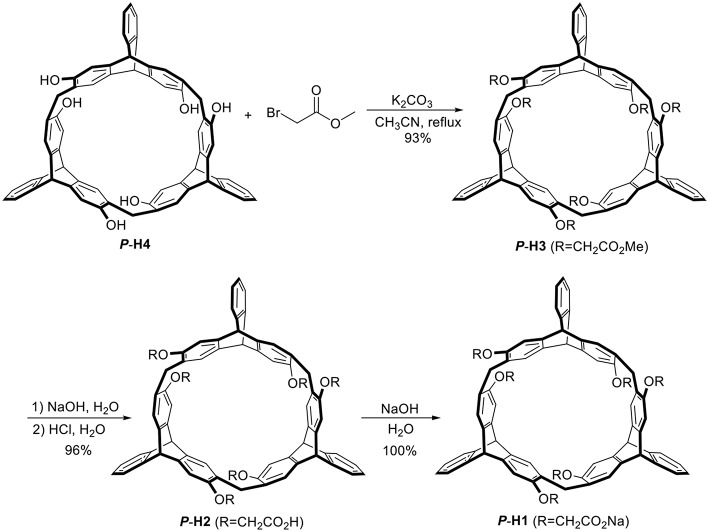
Synthesis of ***P*-H1** and ***M***-**H1**.

As shown in Scheme 1, treatment of ***P***-**H4** and methyl bromoacetate in acetonitrile in the presence of K_2_CO_3_ provided methoxycarbonyl-substituted 2,6-helic[6]arene ***P***-**H3** in 93% yield, which were then followed by the hydrolysis with sodium hydroxide aqueous solution and acidification with hydrochloric acid to give the 2,6-helic[6]arene derivative ***P***-**H2**. Finally, the water-soluble ***P***-**H1** was obtained in 100% yield by treatment of ***P***-**H2** with equivalent sodium hydroxide. According to the same method as described above, 2,6-helic[6]arene derivative ***M***-**H1** could also be conveniently synthesized starting from ***M*-H4**. The new compounds were all characterized by NMR and MS spectra (Figures S1–S4, S12, S13).

### Host-Guest Complexation

**Figure 2:**
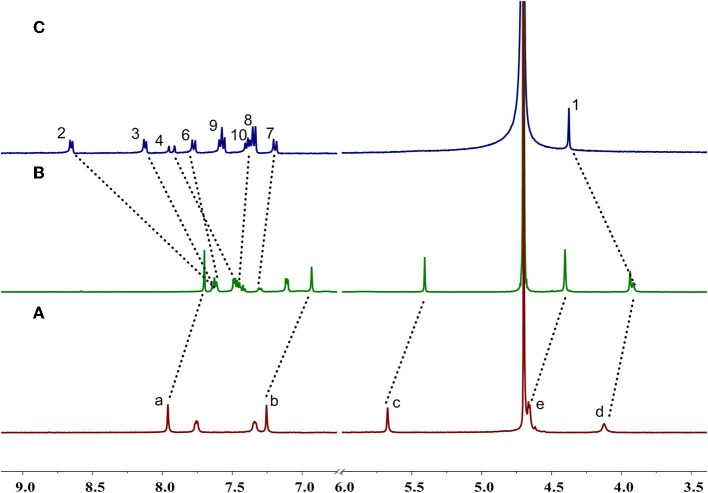
Partial ^1^H NMR spectra (400 MHz D_2_O, 298 K) of **(A)** free ***P***-**H1**, **(B)** ***P***-**H1** with 1.0 equiv. **G**, **(C)** free **G**. [***P***-**H1**] = [**G**] = 2.0 mM.

Enantiomers ***P*-H1** and ***M***-**H1** should show the same host-guest complexation with the guest, so ***P***-**H1** as an example was used to investigate the complexation, which was carried out in solution by ^1^H NMR spectroscopy (Figure 2 and Figure S6 for ***P*-H1·G**, Figures S5, S7 for ***M*-H1·G**). As shown in Figure 2, when 1.0 equiv of host ***P***-**H1** was added into a solution of **G**, significant chemical shift changes of the protons on ***P***-**H1** and **G** appeared. It was found that protons H_1_, H_2_, H_3_, and H_4_ of the guest shifted upfield dramatically by 0.47, 1.02, 0.50, and 0.48 ppm, respectively. While the resonance peaks related to protons H_7_, H_8_, H_10_ showed downfield shifts compared with free guest **G** (Δδ = 0.11, 0.08 and 0.06 ppm, respectively). Moreover, the signals of protons H_a_, H_b_, H_c_, and H_d_ on ***P***-**H1** also showed upfield shifts due to the host-guest interaction. These observations suggested that host ***P***-**H1** could form 1:1 stable complex with guest **G**, and the complexation between ***P***-**H1** and **G** was a fast exchange process on the NMR spectroscopic timescale. From the 2D ROESY spectrum of a solution of 2.0 mM ***P***-**H1** and 2.0 mM **G** (Figures S10, S11), correlations were observed between protons H_2_, H_3_ of guest **G** and protons H_a_ on ***P***-**H1**, which further indicated that in the complex, the methylpyridinium group of **G** was located inside the cavity of ***P***-**H1**, while the benzene ring connected with the double bond of **G** might be located outside the cavity. Furthermore, the electrospray ionization (ESI) mass spectra confirmed the formation of the 1:1 complex between ***P***-**H1** and **G** (Figures S14, S15) as well, in which the signal corresponding to [***P***-**H1·G**-6Na+3H-I]^2+^ was monitored at *m*/*z* 801.24776. To quantitatively investigate the complexation between ***P***-**H1** and **G**, isothermal titration calorimetry (ITC) experiments were then carried out in aqueous solution (Figure S16). Consequently, it was found that the association constant (*K*_a_) for 1:1 complex ***P***-**H1·G** was determined to be (3.84 ± 0.24) × 10^5^ M^−1^. Similarly, ***M***-**H1** could also form 1:1 stable complex with guest **G** in water, and the *K*_a_ value for the 1:1 complex ***M***-**H1·G** was determined to be (3.21 ± 0.23) × 10^5^ M^−1^.

UV/Vis and fluorescence experiments were also performed to investigate the host-guest complexation. As shown in Figure S17, the absorption and emission spectra of 2.00 × 10^−5^ M **G** in aqueous solution exhibited an absorption maximum at 450 nm and a weak emission band at about 600 nm, respectively. When equimolar ***P***-**H1** was added into the solution of **G**, distinct bathochromic shifts of absorption maximum occurred from 450 to 475 nm, while a new emission band centered at 618 nm appeared. Probably due to the cavity inclusion and rotation restriction of **G**, the ACQ effects of **G** were avoided and an intensive emission signal was observed in the aqueous solution of ***P***-**H1·G**.

**Figure 3:**
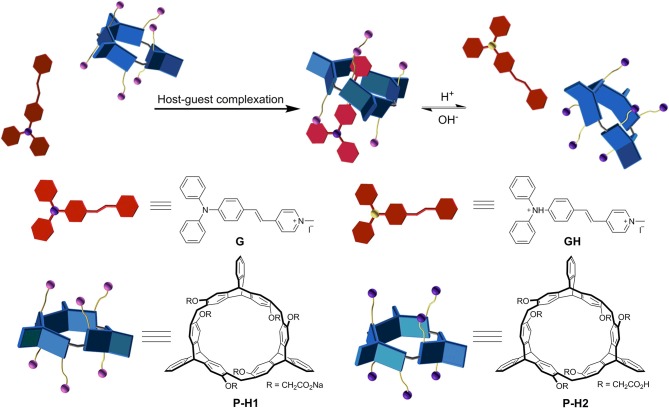
Cartoon representation of pH responsive complex ***P***-**H1·G**.

Since ***P***-**H1** contained six carboxyl groups, we further explored the acid/base controlled complexation of ***P***-**H1·G** by ^1^H NMR spectroscopy. As shown in Figures S8, S9, upon the addition of an aqueous DCl solution into complex ***P***-**H1·G** in D_2_O, the carboxylate groups in ***P***-**H1** were acidified into carboxylic acids, and the protonated host was then precipitated from the solution. Although the guest was also protonated and possessed good water solubility, most of them might be absorbed by the ***P***-**H1** sediment, leading to no signals in the spectrum. Followed by adding an excess of NaOD aqueous solution to the above system, the protonated host dissolved in the solution, and the proton signals of complex ***P***-**H1·G** recovered, which suggested that complex ***P***-**H1·G** formed again. These results indicated that the acid/base stimuli-responsive complexation between ***P***-**H1** and **G** could be efficiently controlled (Figure 3).

### Morphology of the Aggregates

**Figure 4:**
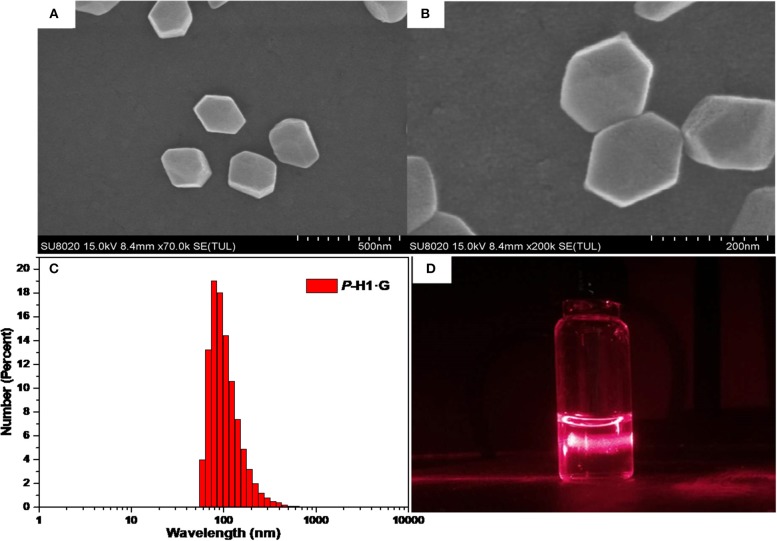
**(A)** SEM image, **(B)** enlarged SEM image, **(C)** hydrodynamic diameter measured by DLS and **(D)** Tyndall effect of the self-assembled ***P***-**H1·G** ([***P***-**H1·G**] = 0.6 mM in H_2_O, pH 7.00).

Based on the formation of complex ***P***-**H1·G**, we further constructed the supramolecular assemblies in water by the reported method (Li et al., 2018b). Consequently, 100 or 600 μL aqueous solution of ***P***-**H1·G** (5 mM) was rapidly injected into 9 mL of water/THF 2:1 (*v*/*v*) solution under ultrasonic condition. After the ultrasound was sustained for 5 min, the solution was bubbled by Ar for about 1 h to remove the THF. Then, by continual bubbling, the obtained solution was heated to 100°C until 5 mL of water remained. Thus, the uniformly distributed self-assemblies in H_2_O were obtained with 0.1 and 0.6 mM, respectively. To study the topological influence of ***P***-**H1** and **G** on the self-assemblies, the similar assembled experiments for free ***P***-**H1** and free **G** were also carried out, respectively. Scanning electron microscopy (SEM) and dynamic light scattering (DLS) were used to investigate the assembled structures of ***P***-**H1·G**. SEM images of the assembled ***P***-**H1·G** (0.6 mM) showed hexagon nanostructures with diameters of about 160 nm (Figures 4A,B). Meanwhile, DLS data showed that the assemblies possessed an average hydrodynamic diameter of 180.4 nm in solution with an obvious Tyndall effect (Figures 4C,D). For the assemblies from the 0.1 mM complex, rectangular nanosheets with the length ranging from 100 to 150 nm were found, which were in agreement with the DLS results (Figures S21–S23). Comparatively, the morphologies of both ***P***-**H1** and **G** showed strip-like structures with a length of several micrometers (Figure S18), which were distinctly different from that of complex ***P***-**H1·G**. For the assemblies of ***M***-**H1·G** formed under the same conditions, similar nanostructures to complex ***P***-**H1·G** were obtained (Figures S20, S24). These results suggested that the macrocyclic compounds have a dramatic influence on the spatial alignment of **G**, and the morphological modulation of the assemblies from the complex could also be realized by simply tuning the concentration of host-gust complex.

Since complex ***P***-**H1·G** could be easily destroyed by acid (Zhang et al., 2017b), we found that when the pH of the assembled solution of ***P***-**H1·G** (0.6 mM) decreased to 3.00, the self-assembly morphology of ***P***-**H1·G** changed from nanohexagon to irregular structures (Figure S19). Moreover, it was also found that when the pH of above system reached 9.00 by adding NaOH solution, irregular aggregates were also generated probably because the existence of excess NaOH could increase the ionic strength of the system and subsequently weaken the host-guest interaction of ***P***-**H1·G** (Figure S19) [17a]. Similarly, the regular nanosheets for the assemblies of 0.1 mM system could also be destroyed upon addition of HCl or NaOH (Figures S21, S22). These results indicated that the self-assembly morphology based on complex ***P***-**H1·G** showed pH responsiveness.

### CD and CPL Properties of the Aggregates

**Figure 5:**
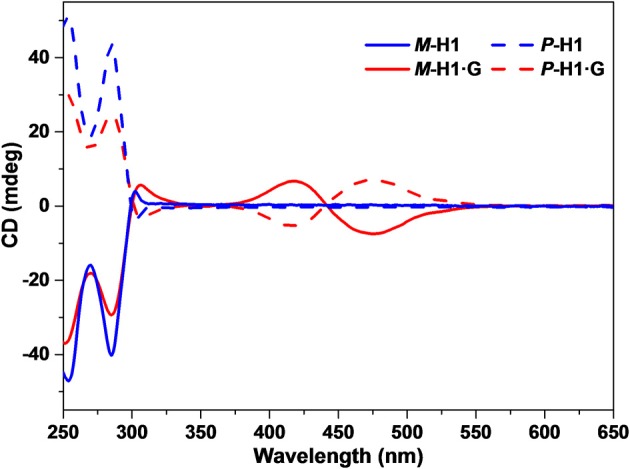
CD spectra of the enantiomeric macrocycles ***P***-**H1**/***M***-**H1** and the self-assemblies from ***P***-**H1·G**/***M***-**H1·G** in H_2_O ([***P***-**H1**] = [***M***-**H1**] = [***P***-**H1·G**] = [***M***-**H1·G**] = 0.1 mM).

Based on the spatial confinement of **G** by the chiral cavity of the macrocycles and strong absorption and emission properties of the complexes, we deduced that the assembled nanostructures could show induced CD and CPL properties, attributed to the chiral transfer from the chiral cavity of ***P***-**H1**/***M***-**H1** to the dye guest **G**. As shown in Figure 5, mirror-image CD signals for ***P***-**H1** and ***M***-**H1** at 285 nm were observed in agreement with their absorption regions. For the assemblies from complexes ***P***-**H1·G**/***M***-**H1·G**, a pair of new mirror-image CD signals at 425 and 475 nm appeared, which could be ascribed to the host-guest complexation induced chiral transfer from the enantiomeric macrocycles to **G**. Moreover, when 0.6 mM solution of assembled complexes ***P***-**H1·G**/***M***-**H1·G** was used, the CD signals enhanced distinctly, suggesting that the system with higher concentration could induce stronger chirality.

**Figure 6:**
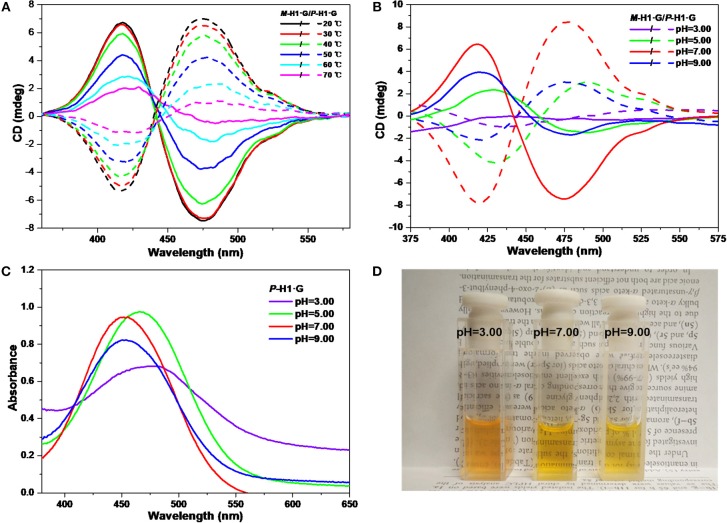
CD spectra of the assemblies from ***P***-**H1·G** and ***M***-**H1·G** by gradually increasing **(A)** the temperature and **(B)** tuning the pH. **(C)** Influence of pH on UV/vis spectra of ***P***-**H1·G** assembled solution. **(D)** Photograph showing color changes with different pH of assembled ***P***-**H1·G** solution. [***P***-**H1·G**] = [***M***-**H1·G**] = [**G**] = 0.1 mM, solvent: H_2_O.

We further explored the CD changes of the assemblies under alternative temperatures and pH values. It was found that with the increase of temperature from 20 to 70°C, the intensity of the mirror-image CD signals induced by the host-guest complexation decreased gradually (Figure 6A). These changes implied that the accelerated rotation and motion of the chiral macrocycle and guest **G** emerged under heating conditions, which resulted in the interruptive chirality transfer. It was also found that when dilute HCl solution was added into the neutral system, the mirror-image CD signals exhibited reduced intensity as well as bathochromic shifts (Figure 6B), indicating that the assemblies dissociated gradually while the host and the guest were protonated. Simultaneously, when pH increased to 9.00, only the weakened CD signal intensities were observed due to no structural change of guest **G**. The pH responsive CD signal changes of the assemblies from the complexes were basically in agreement with the change tendency of UV/vis spectra and color changes in Figures 6C,D.

**Figure 7:**
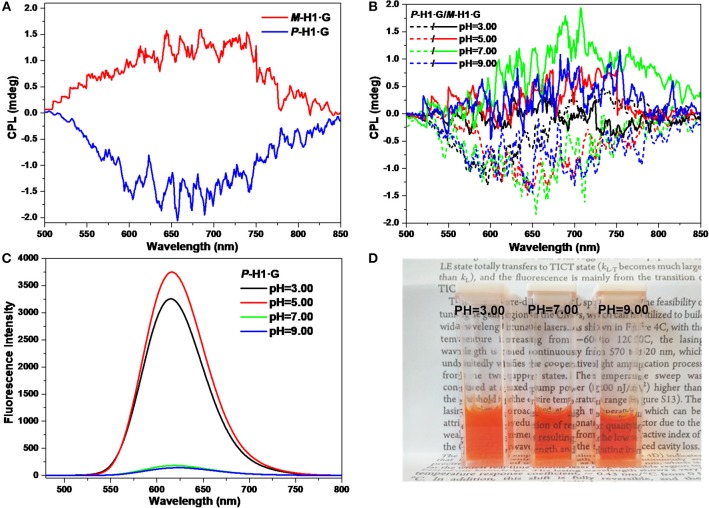
**(A)** CPL spectra of the assemblies from ***P***-**H1·G** and ***M***-**H1·G** in water. **(B)** CPL spectra, **(C)** FL spectra, and **(D)** photograph showing color changes with different pH values of the assemblies from ***P***-**H1·G** and ***M***-**H1·G** in water ([***P***-**H1·G**] = [***M***-**H1·G**] = 0.6 mM, λ_ex_ = 450 nm).

The CPL properties of the assemblies form the complexes were further explored. As shown in Figure 7A, it is found that with chiral microenvironment of the macrocycles and luminophore of the guest, the ***P***-**H1·G**/***M***-**H1·G** assembled solution (0.6 mM) also showed a pair of mirror-image CPL signals ranging from 500 to 850 nm, which might be attributed to the chirality transfer from the macrocycles to guest **G** by the strong host-guest interactions and the well-ordered assembled nanostructures. Correspondingly, the maximum *g*_lum_ values of CPL for ***P***-**H1·G** and ***M***-**H1·G** were determined to be −2.67 × 10^−4^ and 1.48 × 10^−4^ at 655 nm, respectively. Moreover, the pH values dependent on CPL signal changes of the assembled solution could also be found. As shown in Figure 7B, the mirror-image CPL signals weakened under either the acidic or basic conditions. These observations might be due to the destruction of the assemblies from complex ***P***-**H1·G**/***M***-**H1·G** by acid, and the weakened host-guest interactions by base, which were further proved by FL spectra and photograph showing color changes (Figures 7C,D). The acid/base controlled CPL properties of the assemblies could provide an opportunity to further design and construct new chiral assembled materials with responsive properties.

## Conclusion

In summary, we have synthesized a couple of water-soluble chiral 2,6-helic[6]arene derivatives ***P***-**H1** and ***M***-**H1**, and found that they could form 1:1 stable complexes with 4-[(4'-*N, N*-diphenylamino)styryl]-*N*-methylpyridinium iodide in water. Compared with the guest, the host-guest complexes exhibited enhanced fluorescence, which might be attributed to the spatial confinement of the guest and restriction of ACQ effects. Based on the host-guest complexation, the first helic[6]arene-based chiral assemblies were then constructed, and they showed rectangular or hexagonal nanostructures by SEM images. Interestingly, it was found that the assemblies showed clear mirror-image CD and CPL spectra in aqueous solution, which revealed a consecutive chirality transfer from the chiral macrocycles to the achiral guest. Moreover, the assemblies could also show the responsive CD and CPL activities to the pH and temperatures, which would provide an opportunity to further construct new chiral functional materials.

## Data Availability

All datasets generated for this study are included in the manuscript/Supplementary Files.

## Author Contributions

This work was done mainly by YG, and the manuscript was written by YG with the guidance of YH and C-FC.

### Conflict of Interest Statement

The authors declare that the research was conducted in the absence of any commercial or financial relationships that could be construed as a potential conflict of interest. The handling editor declared a shared affiliation, though no other collaboration, with the authors [YG, YH, C-FC] at time of review.
