# Supplementary material for: Construction of Chiral Nanoassemblies Based on Host-Guest Complexes and Their Responsive CD and CPL Properties: Chirality Transfer From 2,6-helic[6]arenes to a Stilbazolium Derivative
Source: Front Chem. 2019 Aug 2;7:543. doi: 10.3389/fchem.2019.00543 (PMC6688524; doi:10.3389/fchem.2019.00543)
Supplement: Supplementary file 1 [file Data_Sheet_1.PDF]

Supporting Information

for

Construction of chiral nanoassemblies based on host-guest complexes and their responsive CD and CPL properties: chirality transfer from 2,6-helic[6]arenes to a stilbazolium derivative

Yan Guo[1,2] Ying Han*[,1] and Chuan-Feng Chen*[1,2]

Address: [1]Beijing National Laboratory for Molecular Sciences, CAS Key Laboratory of Molecular Recognition and Function, Institute of Chemistry, Chinese Academy of Sciences, Beijing 100190, China. [2]University of Chinese Academy of Sciences, Beijing 100049, China.

E-mail: cchen@iccas.ac.cn

Experimental procedures, NMR spectra, mass spectra, photophysical properties, SEM images and DLS spectra

Table of Contents

1. Experimental section S2
2. NMR spectra of new compounds and host-guest complexes S3
3. Mass spectra of host-guest complexes S9
4. ITC experiment of host-guest complexes S10
5. Photophysical properties of host-guest complexes S11
6. SEM images of nanoassemblies S13
7. DLS measurements of nanoassemblies S16
8. References S17

1. Experimental section

General methods All reagents and solvents were purchased from commercial sources and used without further purification. ^1H, ^{13}C and 2D ROESY NMR spectra were recorded on AVIII 400 and 500 MHz NMR spectrometers. High resolution mass spectra were measured on a Thermo Fisher® Exactive high resolution LC-MS spectrometer. ITC experiments were proceeded on GE® MicroCal Auto-ITC 200. UV-Vis spectra were recorded on PerkinElmer® UV/Vis/NIR spectrometer (Lambda 950), and the fluorescence spectra were recorded on HITACHI® F-7000 Fluorescence Spectrometer. CD spectra was recorded on a JASCO J815 spectropolarimeter, and CPL was performed with a JASCO CPL-300 spectrometer. SEM images were obtained on Hitachi® SU-8020. DLS measurements were implemented on Zetasizer Nano ZS ZEN3600 of Malvern Instruments Ltd. The hosts ***P*-H2**/***M*-H2** and guest **G** were prepared according to the same method as literature procedure [1,2].

Synthesis of the hosts

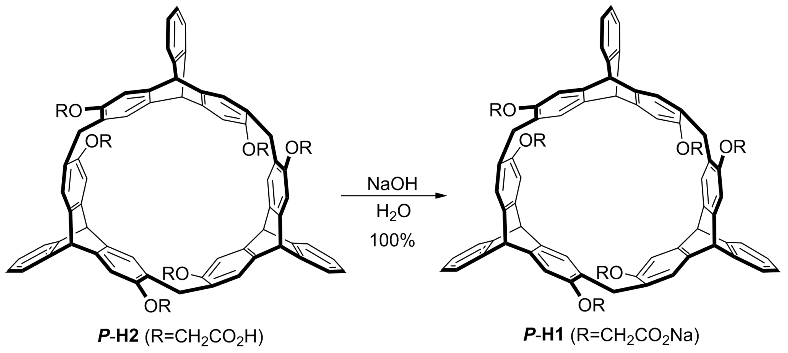

Scheme 1: Synthesis of ***P*-H1** and ***M*-H1**.

***P*-H1**: To the 2,6-helic[6]arene derivative ***P*-H2** (110 mg, 0.089 mmol) was added 10 mL solution of sodium hydroxide (1.0 equiv.), and the mixture was then stirred at room temperature for 4 h. The solvent was removed by rotary evaporation to give

water-soluble host ***P*-H1** (121 mg, 100 %) as a beige solid. M.p. >300 °C. ^{1}H NMR (400 MHz, D_2O, 298 K): δ 7.73 (s, 6H), 7.58-7.56 (m, 7H), 7.18-7.16 (m, 6H), 7.04 (s, 6H), 5.49 (s, 6H), 4.59-4.55 (m, 12H), 4.00 (s, 6H). ^{13}C NMR (126 MHz, D_2O, 298 K): δ 176.3, 155.4, 149.0, 147.0, 139.7, 128.5, 128.4, 127.0, 125.7, 111.6, 71.0, 54.4, 30.7. HRMS (ESI): *m*/*z* calcd for [***P*-H1**-6Na+3H]$^{3-}$ $C_{75}H_{51}O_{18}^{3-}$, 413.1031; found 413.1026.

M*-H1**: Water-soluble host ***M*-H1** was synthesized by the same method as ***P*-H1**. M.p. >300 °C. ^{1}H NMR (400 MHz, D_2O, 298 K): δ 7.73 (s, 6H), 7.57-7.54 (m, 6H), 7.17-7.15 (m, 6H), 7.03 (s, 6H), 5.47 (s, 6H), 4.57-4.53 (m, 12H), 3.99 (s, 6H). ^{13}C NMR (126 MHz, D_2O, 298 K): δ 176.3, 155.4, 149.0, 147.0, 139.7, 128.5, 128.4, 127.0, 125.7, 111.6, 71.0, 54.4, 30.7. HRMS (ESI): *m*/*z* calcd for [M*-H1**-6Na+3H]$^{3-}$ $C_{75}H_{51}O_{18}^{3-}$, 413.1031; found 413.1025.

2. NMR spectra of new compounds and host-guest complexes

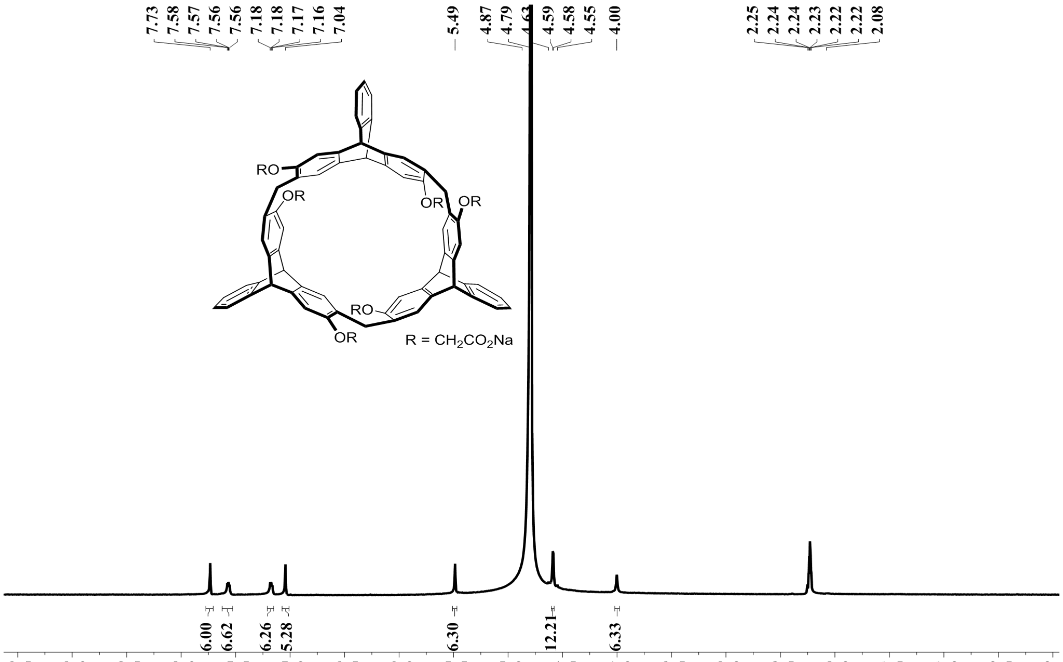

Figure S1: ^{1}H NMR spectra of ***P*-H1** (400 MHz, D_2O, 298 K).

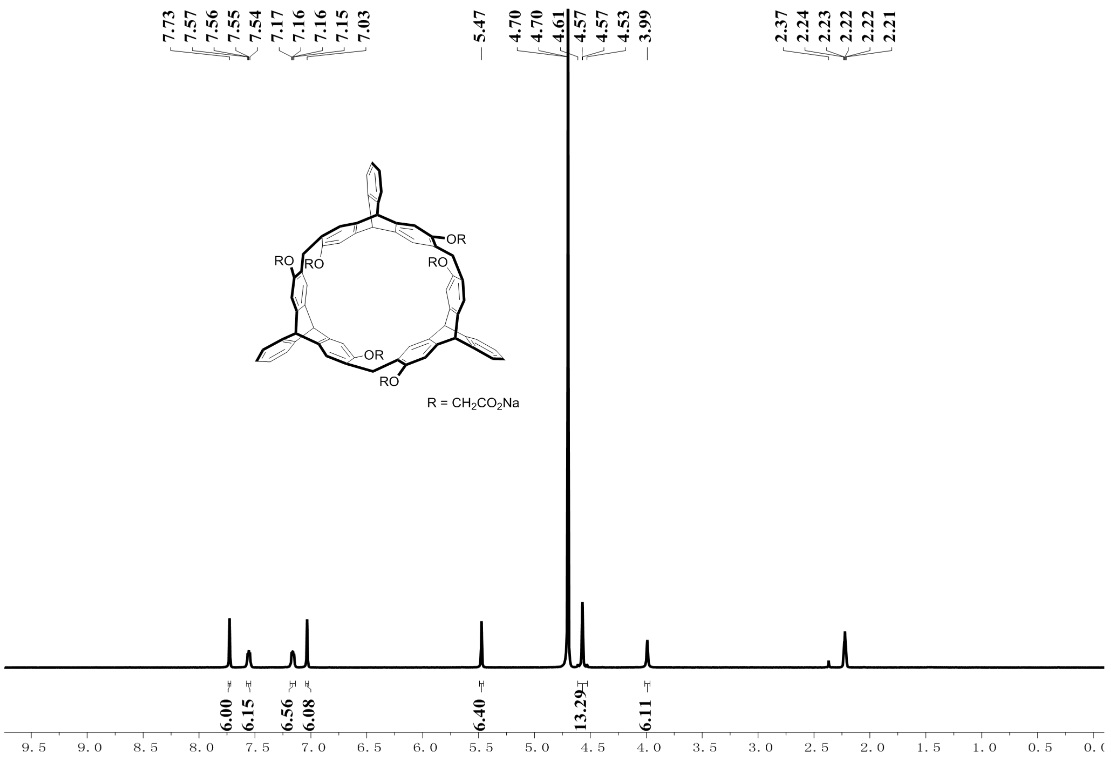

Figure S2: ^{1}H NMR spectra of ***M*-H1** (400 MHz, D_2O, 298 K)

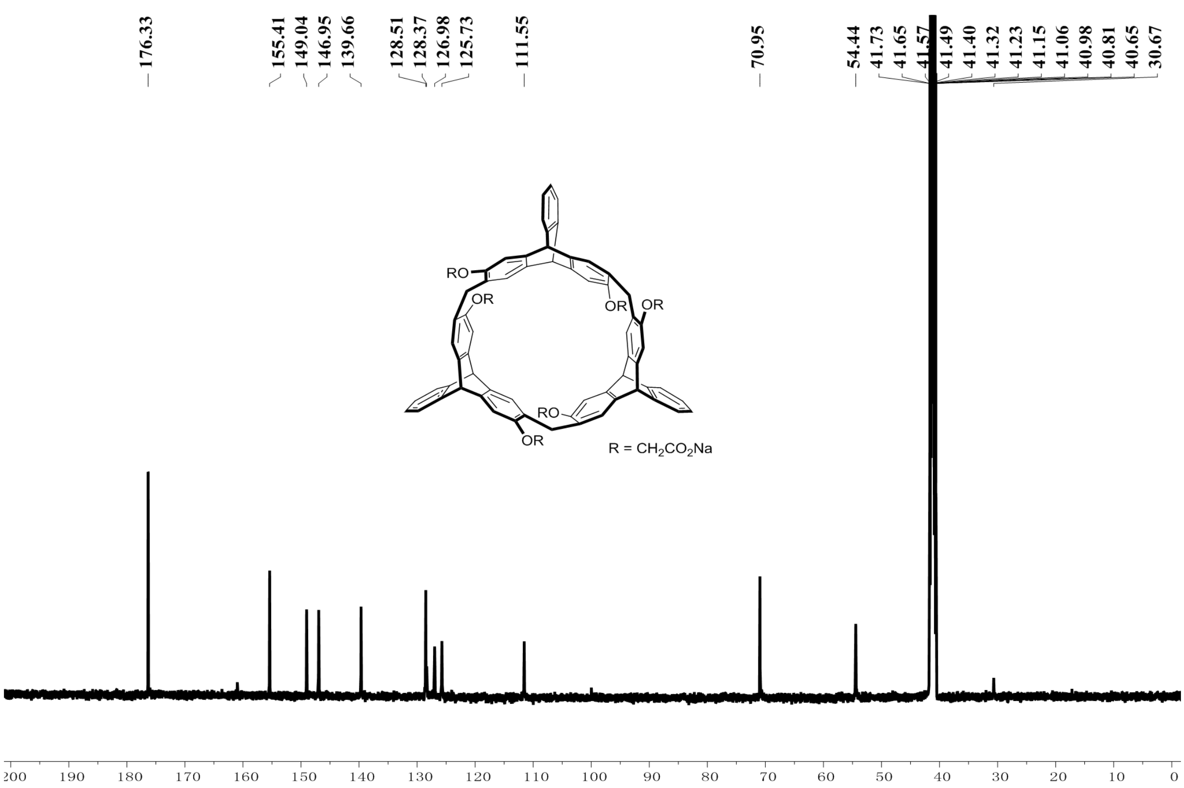

Figure S3: ^{13}C NMR spectra of ***P*-H1** (126 MHz, D_2O, 298 K).

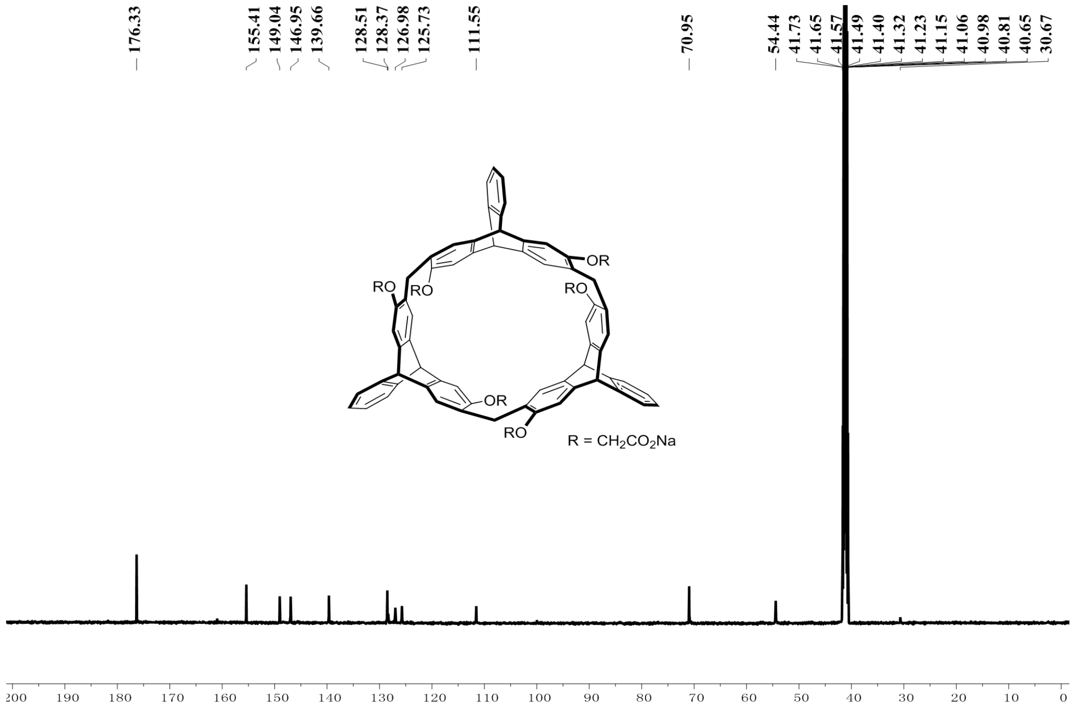

Figure S4: ^{13}C NMR spectra of ***M*-H1** (126 MHz, D_2O, 298 K).

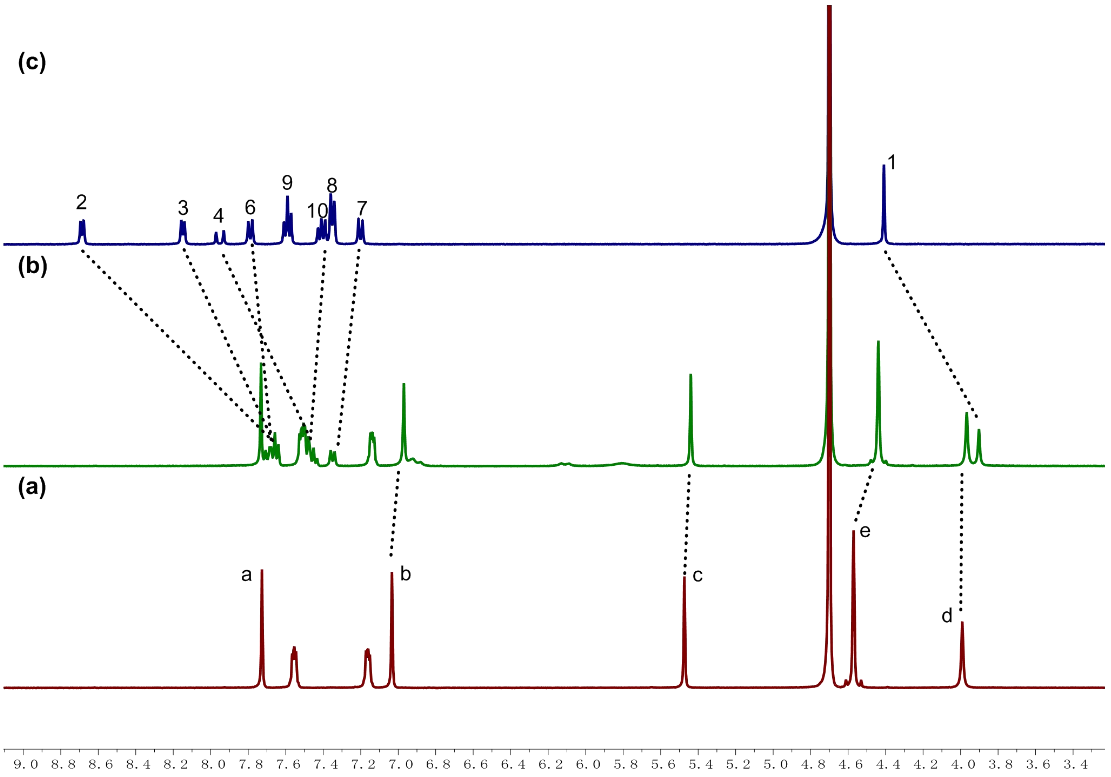

Figure S5: Partial ^{1}H NMR spectra (400 MHz D_2O, 298 K) of (a) free ***M*-H1**, (b) ***M*-H1** with 1.0 equiv. **G**, (c) free **G**. [***M*-H1**] = [**G**] = 2.0 mM.

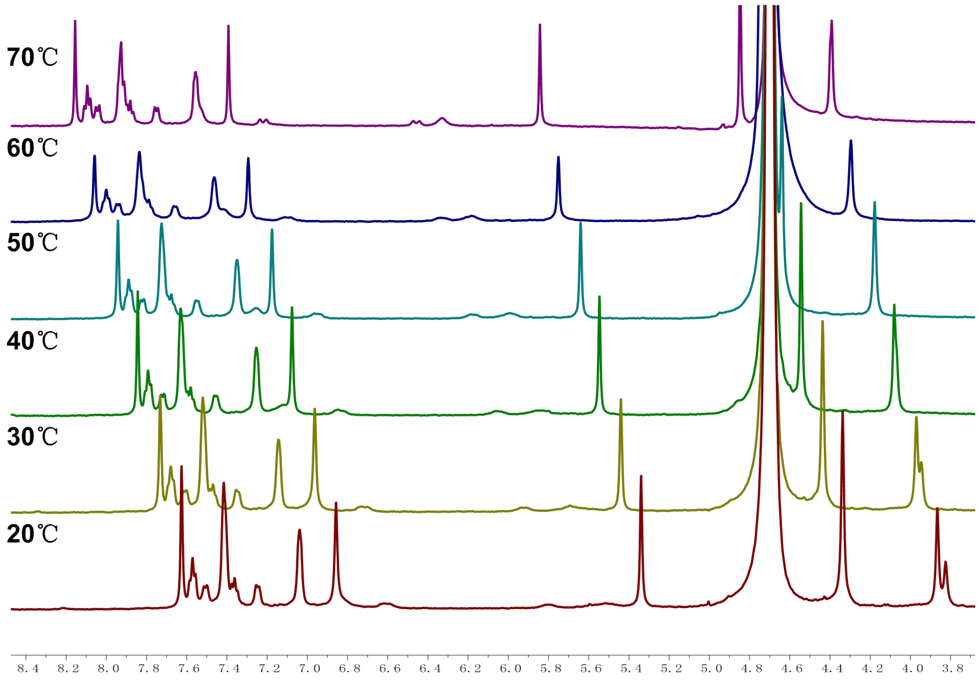

Figure S6: ^{1}H NMR spectra of ***P*-H1·G** complex depending on temperature (500 MHz, D_2O, [***P*-H1**] = [**G**] = 2.0 mM).

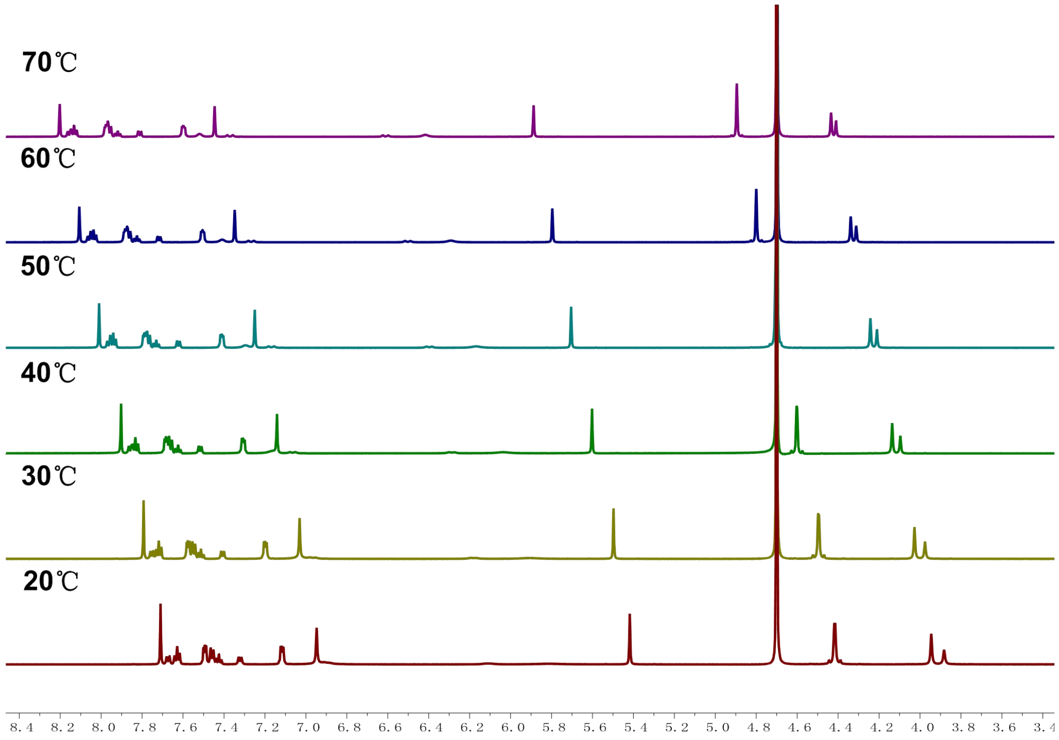

Figure S7: ^{1}H NMR spectra of ***M*-H1·G** complex depending on temperature (600 MHz, D_2O, [***M*-H1**] = [**G**] = 2.0 mM).

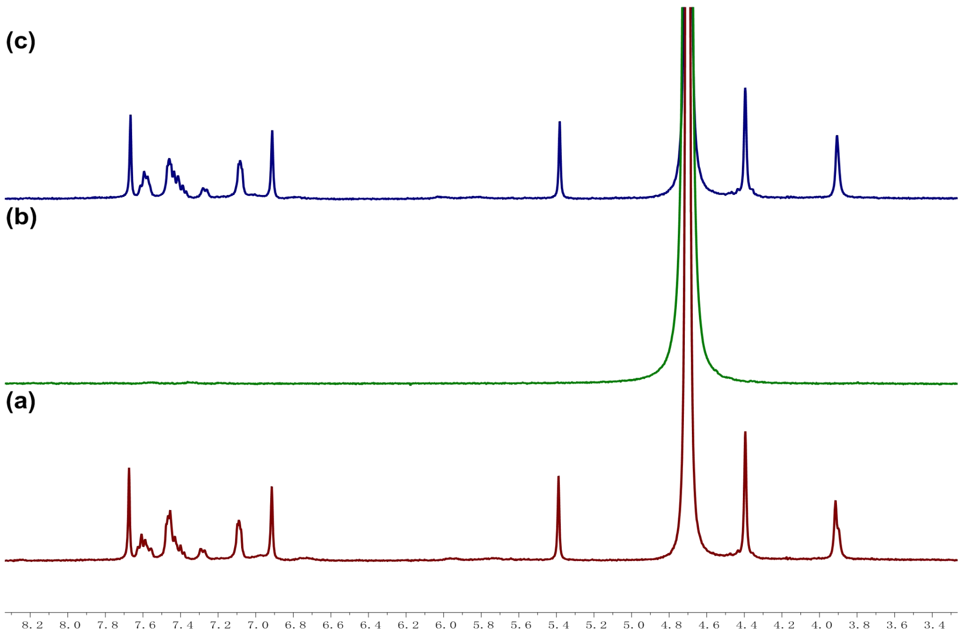

Figure S8: ^{1}H NMR spectra (400 MHz, D_2O, 298 K) of (a) ***P*-H1·G** complex upon addition of (b) 1.0 μL aqueous DCl solution (20 wt%) and (c) 1.5 μL aqueous NaOD solution (40 wt%). ([***P*-H1**] = [**G**] = 2.0 mM)

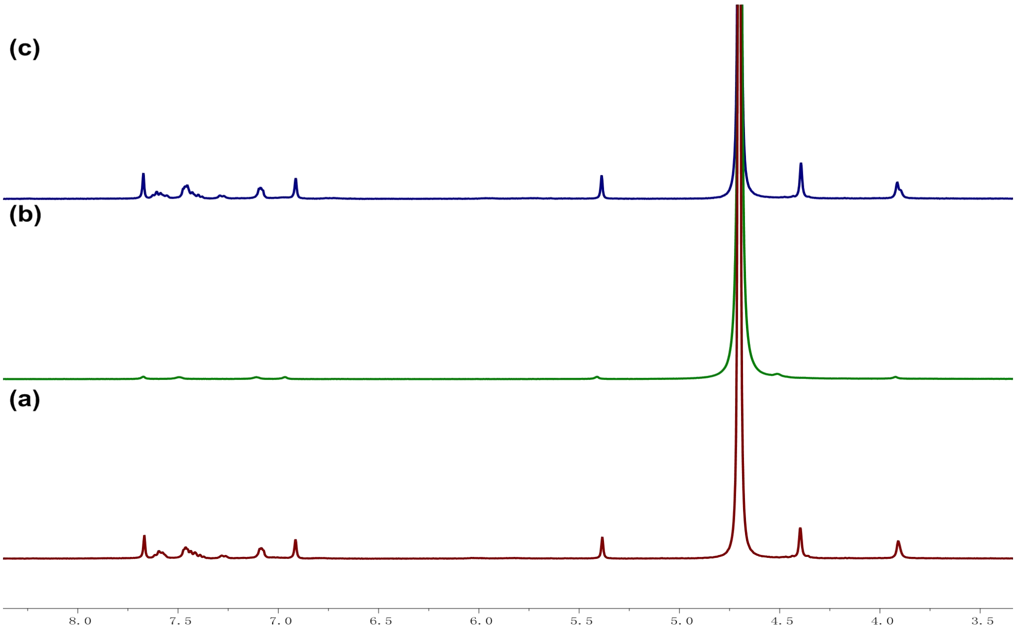

Figure S9: ^{1}H NMR spectra (400 MHz, D_2O, 298 K) of (a) ***M*-H1·G** complex upon addition of (b) 1.0 μL aqueous DCl solution (20 wt%) and (c) 1.5 μL aqueous NaOD solution (40 wt%). ([***M*-H1**] = [**G**] = 2.0 mM)

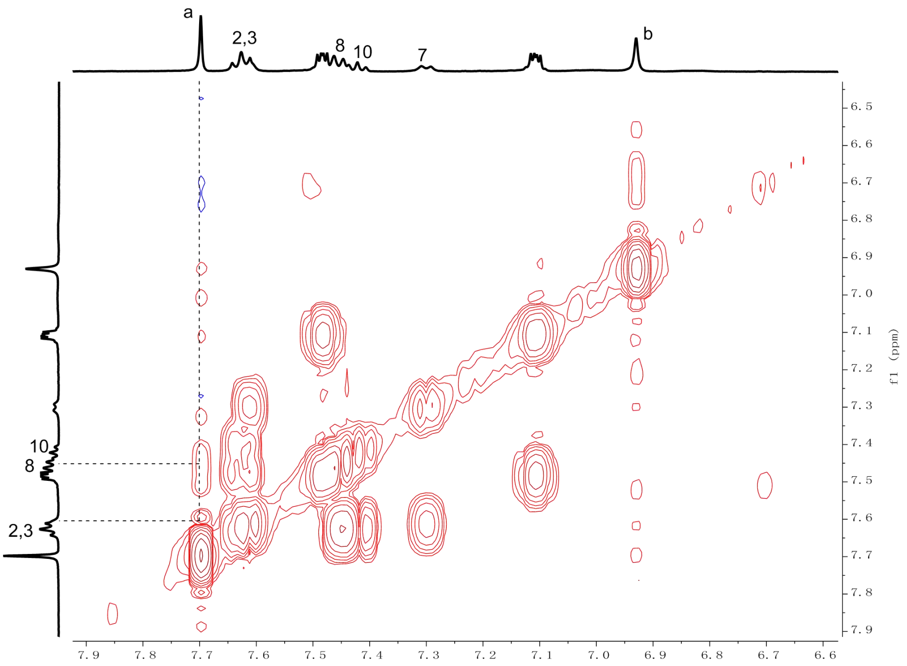

Figure S10: 2D ROESY spectra of ***P*-H1·G** complex (500 MHz, D_2O, 298 K, [***P*-H1**] = [**G**] = 2.0 mM).

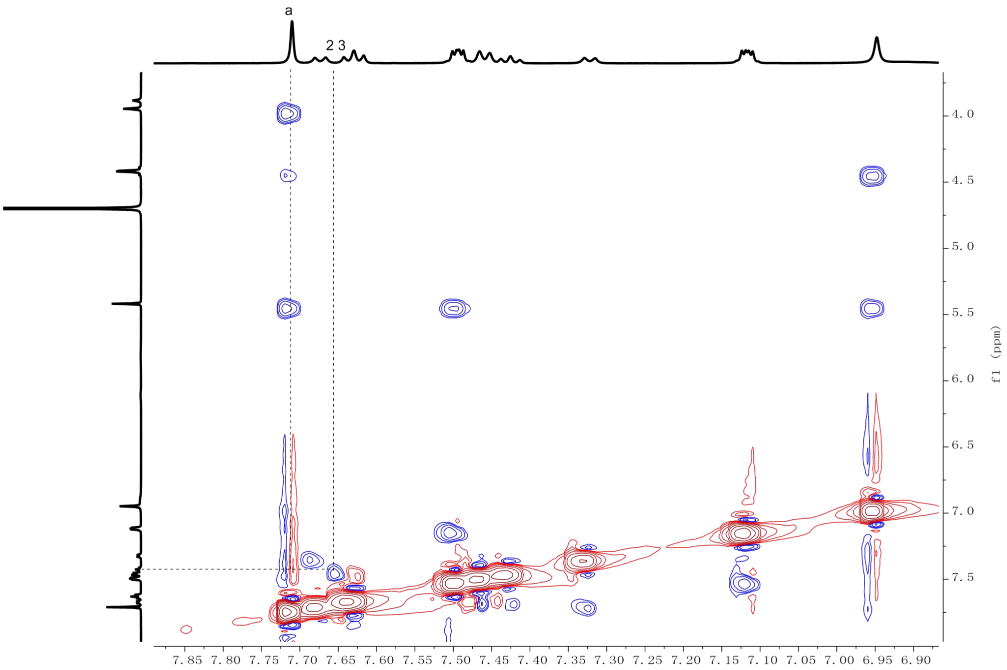

Figure S11: 2D ROESY spectra of ***M*-H1·G** complex (600 MHz, D_2O, 298 K, [***M*-H1**] = [**G**] = 2.0 mM).

3. Mass spectra of new compounds and host-guest complexes

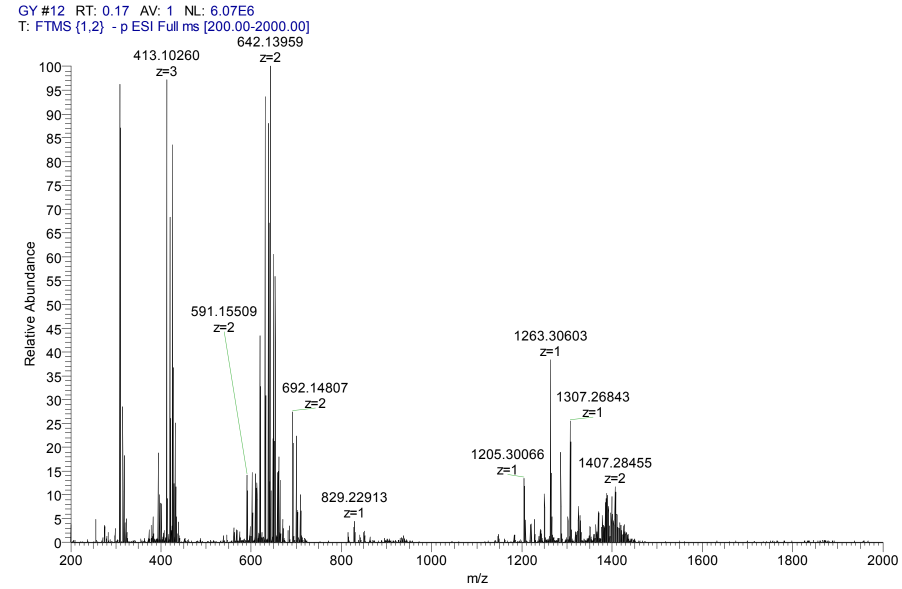

Figure S12: Electrospray ionization mass spectrum of ***P*-H1**.

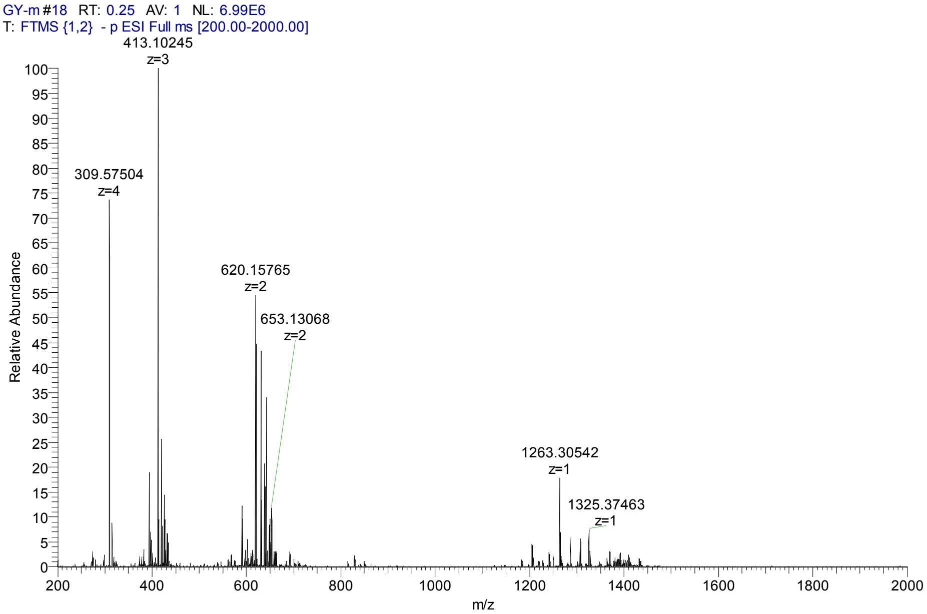

Figure S13: Electrospray ionization mass spectrum of ***M*-H1**.

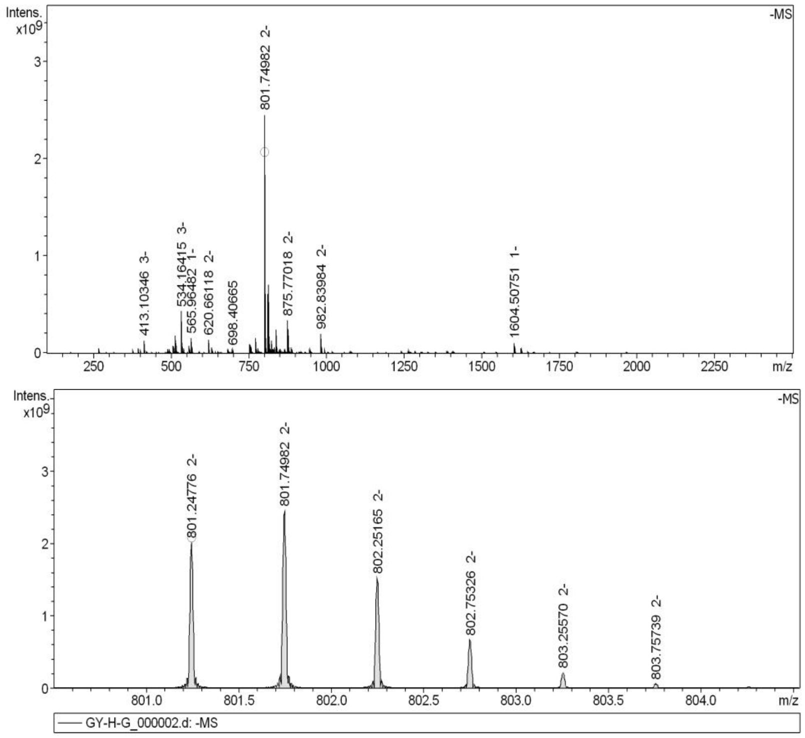

Figure S14: Electrospray ionization mass spectrum of ***P*-H1·G** complex. Assignment of the peak: *m/z* 801.24776 [***P*-H1·G**-6Na+3H-I]$^{2-}$.

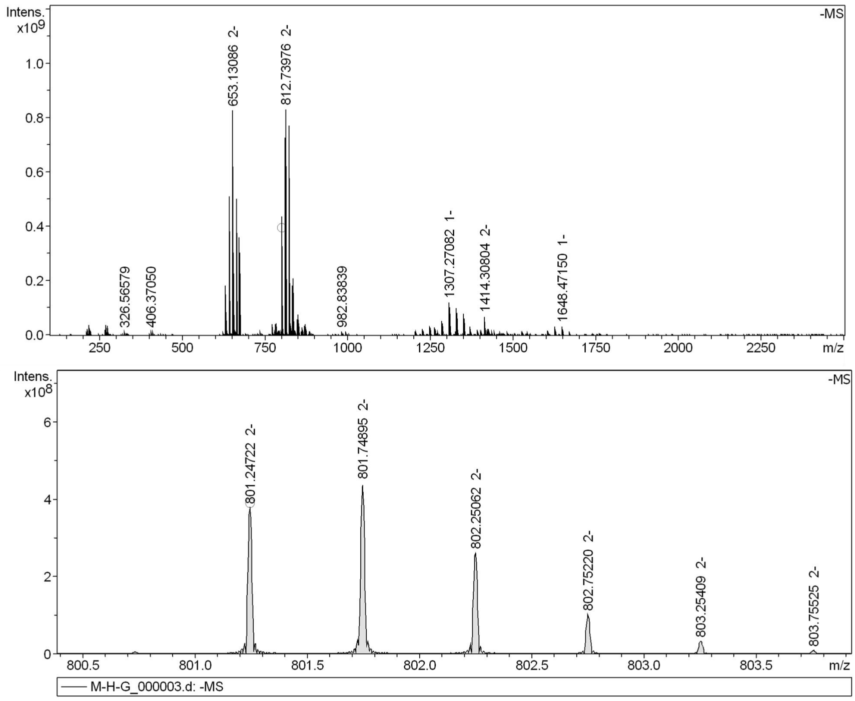

Figure S15: Electrospray ionization mass spectrum of ***M*-H1·G** complex. Assignment of the peak: *m/z* 801.24722 [***M*-H1·G**-6Na+3H-I]$^{2-}$.

4. ITC experiment of host-guest complex

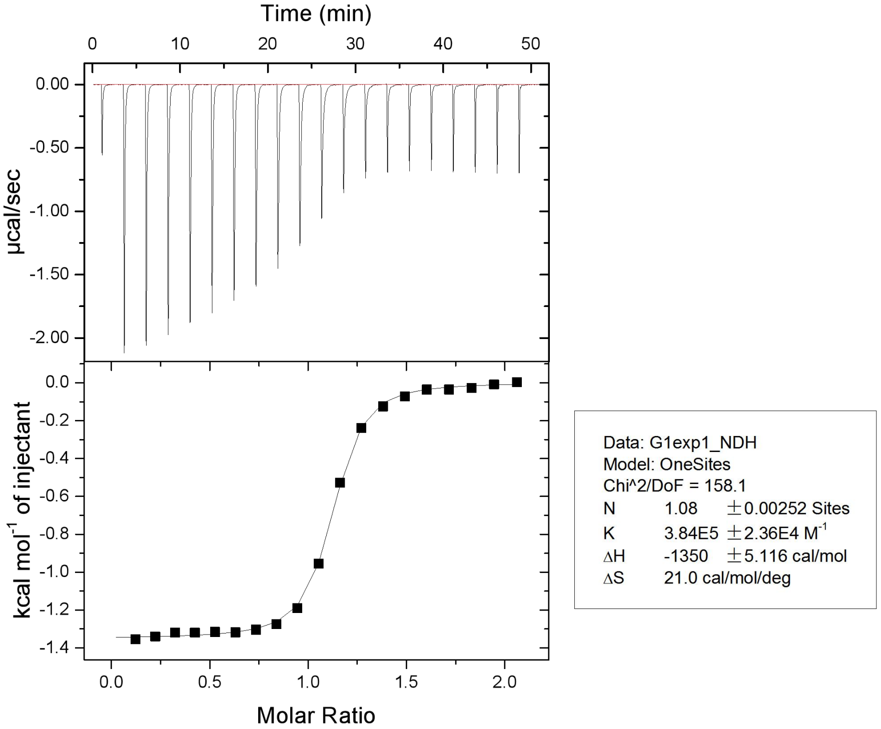

Figure S16: Microcalorimetric titration of **G** with ***P*-H1** in water mixed with 2% DMSO at 298.15 K. (Top) Raw ITC data for 19 sequential injections (2.00 μL per injection) of a **G** solution (2.00 mM) into a ***P*-H1** solution (0.200 mM). (Bottom) Net reaction heat obtained from the integration of the calorimetric traces.

5. Photophysical properties of host-guest complex

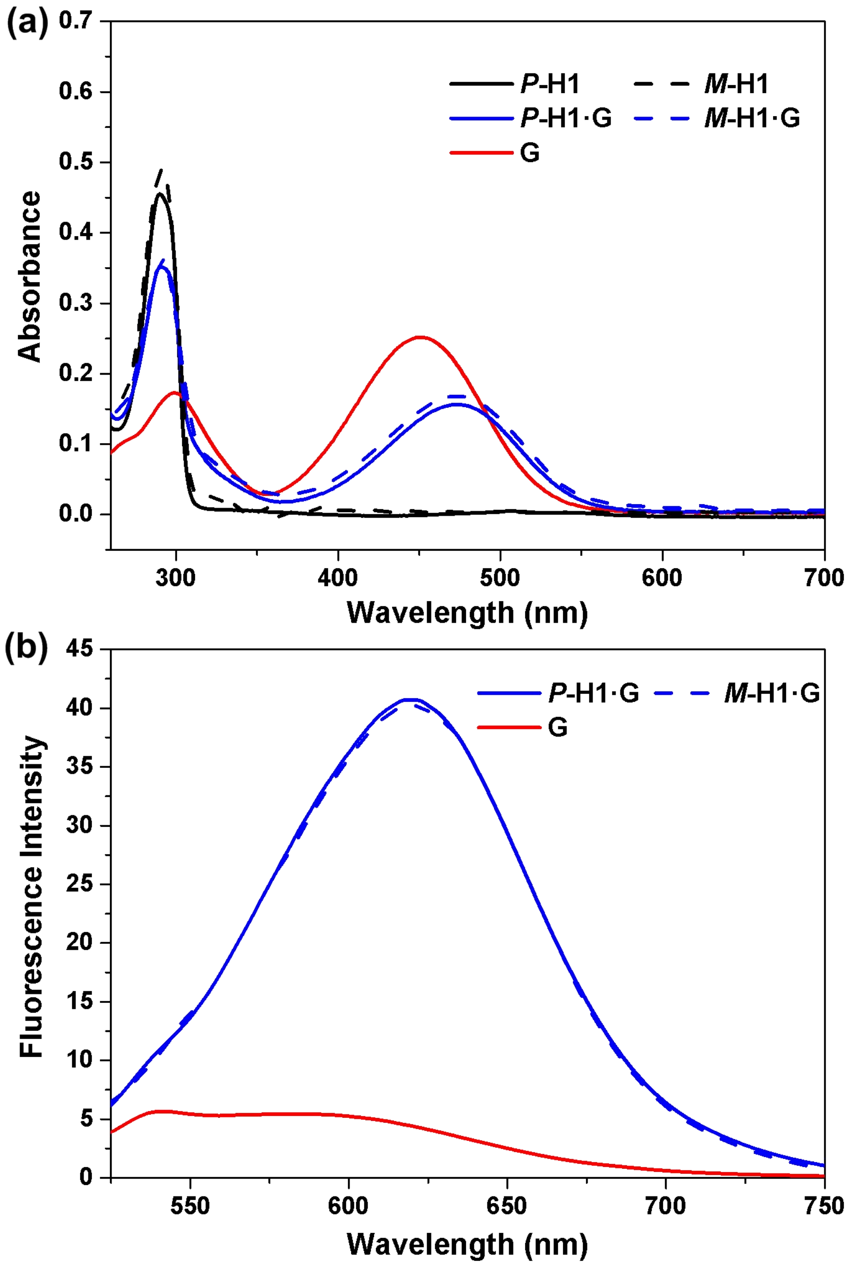

Figure S17: (a) Absorbance spectra of ***P*-H1**/***M*-H1**, ***P*-H1·G**/***M*-H1·G** and **G** in aqueous solution. (b) Fluorescence spectra of **G** without and in the presence of 1.0 equiv of ***P*-H1**/***M*-H1** in aqueous solution ([***P*-H1**] = [***M*-H1**] = [***P*-H1·G**] = [***M*-H1·G**] = [**G**]= 2.00 × 10^{-5} M, λ_{ex} = 450 nm).

6. SEM images of nanoassemblies

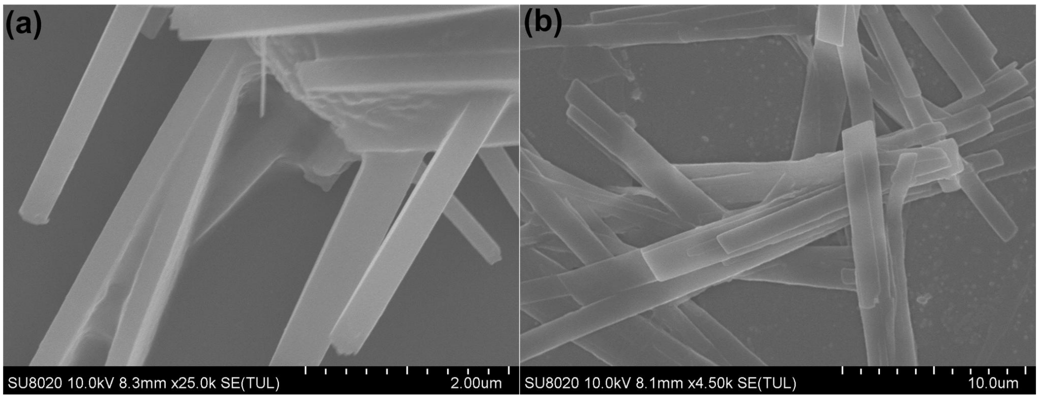

Figure S18: SEM images of (a) ***P*-H1** and (b) **G** aggregates ([***P*-H1**] = [**G**] = 0.6 mM, solvent: H_2O, pH = 7.00).

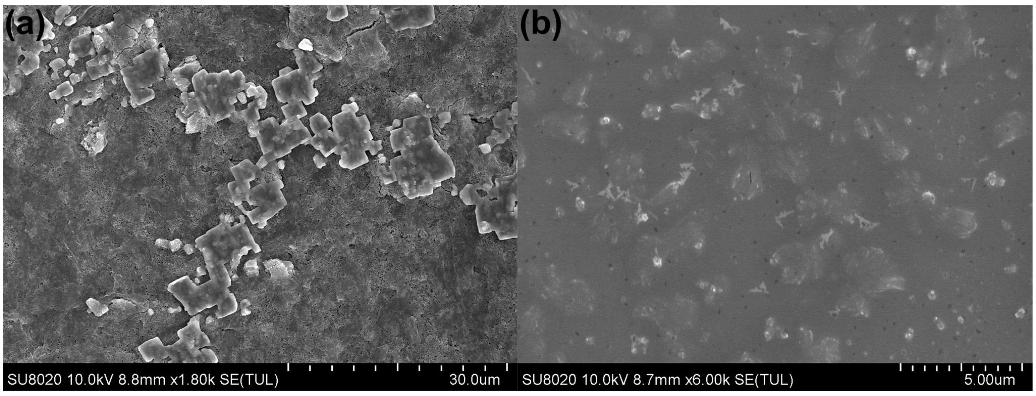

Figure S19: SEM images of assembled ***P*-H1·G** complex (a) with pH = 3.00 and (b) 9.00 ([***P*-H1·G**] = 0.6 mM, solvent: H_2O).

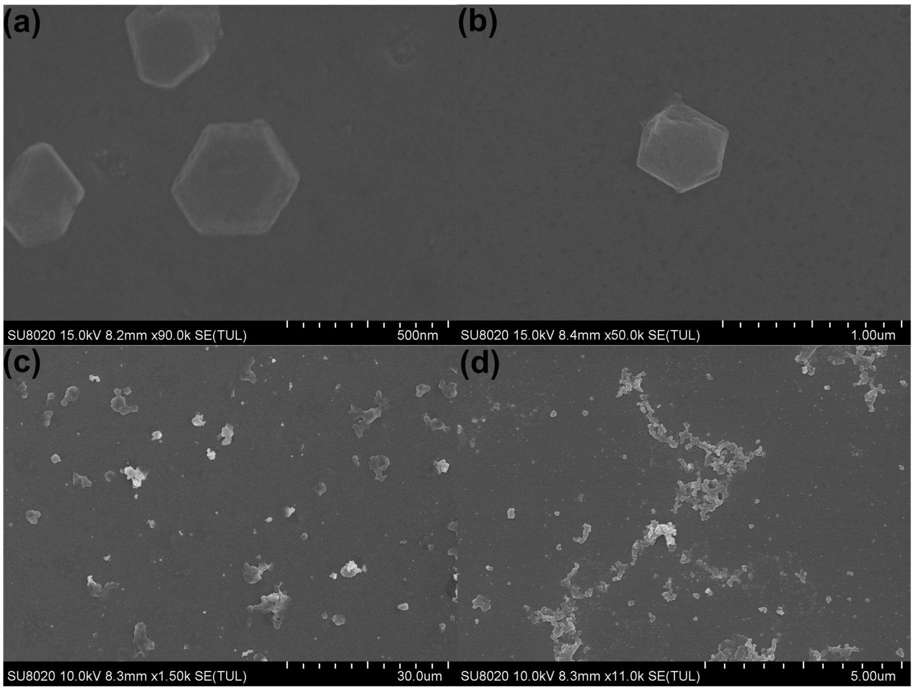

Figure S20: SEM images of assembled ***M*-H1·G** complex (a), (b) in neutral solution and (c), (d) with pH = 3.00 and 9.00, respectively ([***M*-H1·G**] = 0.6 mM, solvent: H_2O).

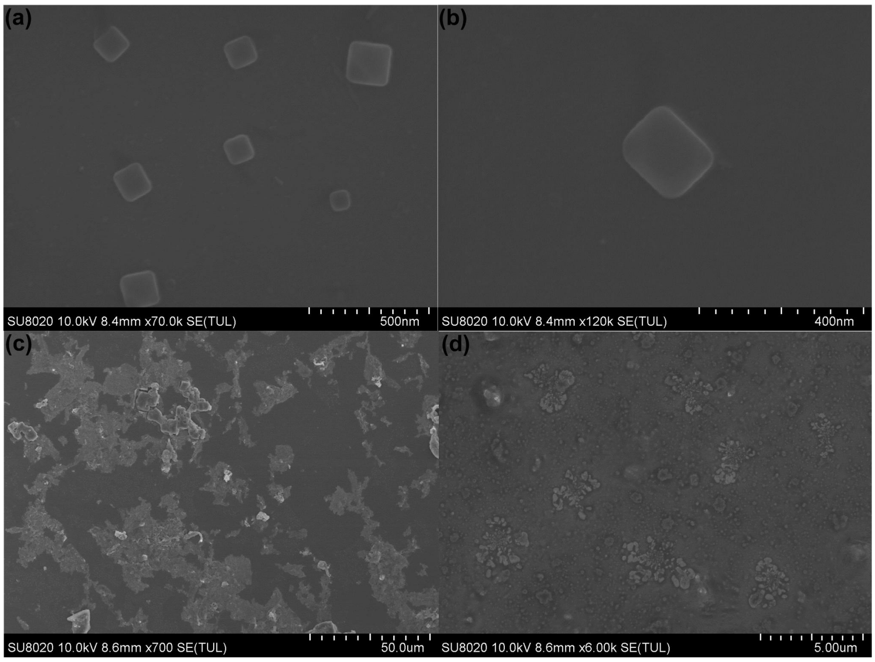

Figure S21: SEM images of assembled (a), (b) ***P*-H1·G** complex in neutral solution and (c), (d) with pH = 3.00 and 9.00, respectively ([***P*-H1·G**] = 0.1 mM, solvent: H_2O).

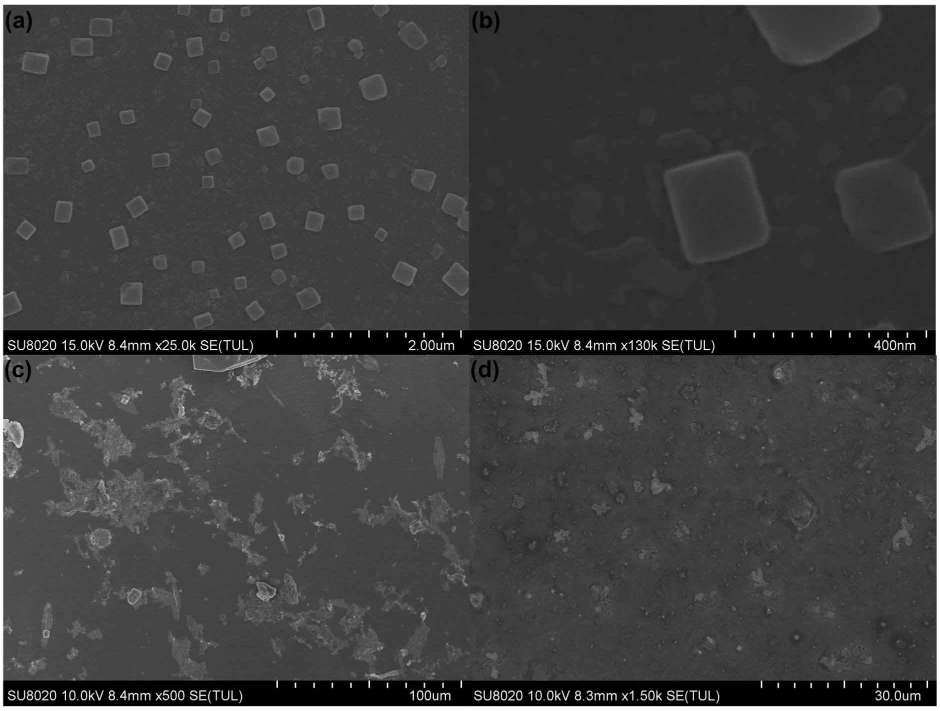

Figure S22: SEM images of assembled (a), (b) ***M*-H1·G** complex in neutral solution and (c), (d) with pH = 3.00 and 9.00, respectively ([***M*-H1·G**] = 0.1 mM, solvent: H_2O).

7. DLS spectra of nanoassemblies

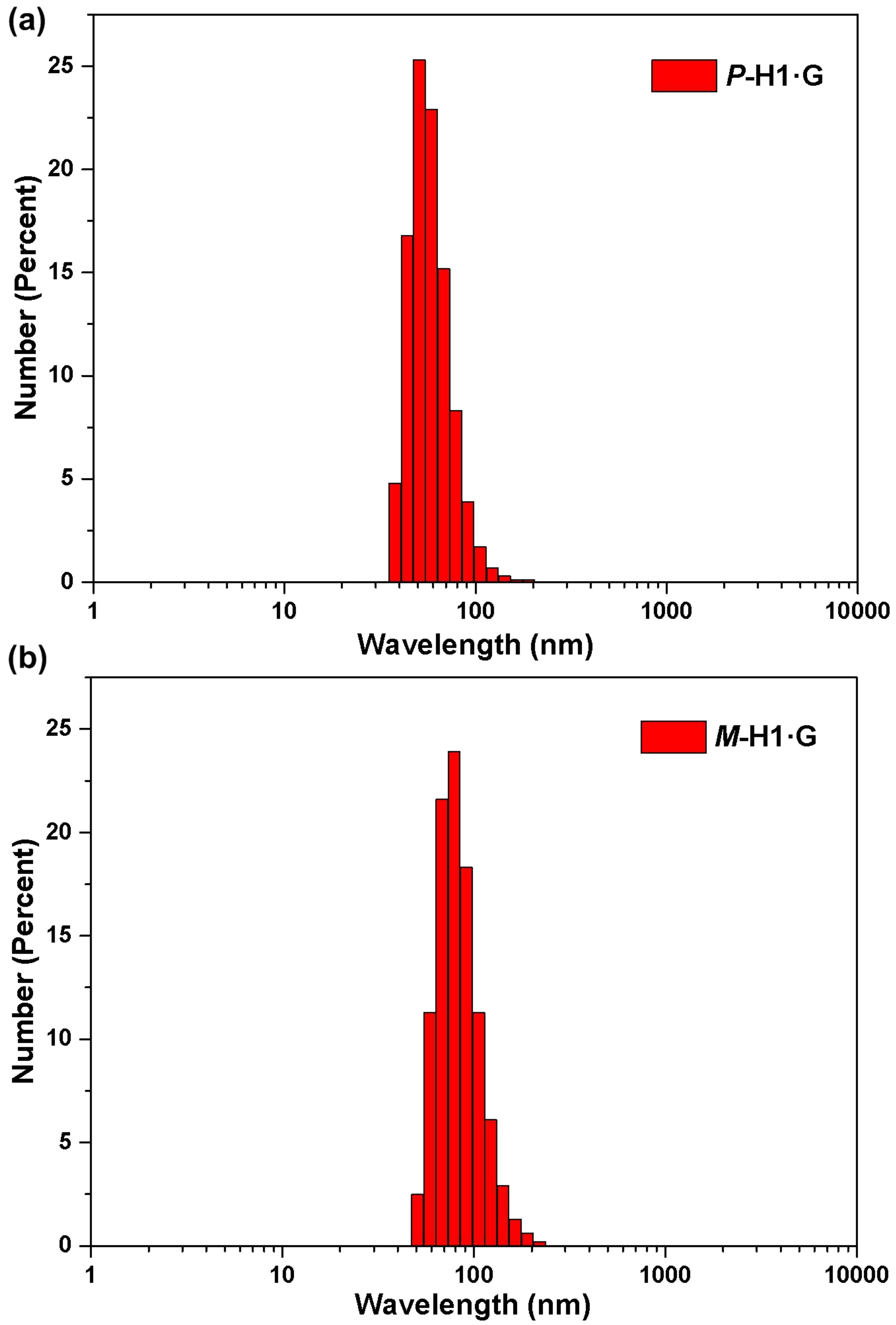

Figure S23: DLS images of assembled (a) ***P*-H1·G** complex (b) ***M*-H1·G** complex ([***P*-H1**·G] = [***M*-H1·G**] = 0.1 mM, solvent: H_2O, pH = 7.00).

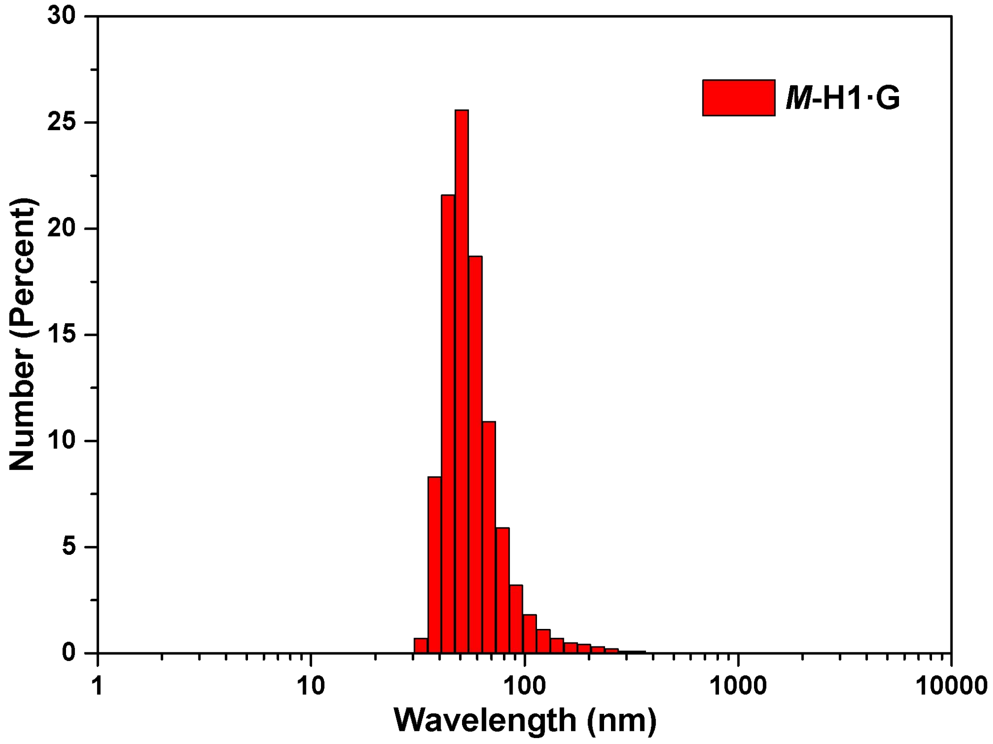

Figure S24: DLS images of assembled ***M*-H1·G** complex ([***M*-H1·G**] = 0.6 mM, solvent: H_2O, pH = 7.00).

8. References

[1] Zhang, G.-W.; Han, Y.; Wang, Y.-L.; Chen, C.-F. *Chem. Commun.* **2017**, *53*, 10433-10436.

[2] Wang, X.-M.; Zhou, Y.-F.; Yu, W.-T.; Wang, C.; Fang, Q.; Jiang, M.-H.; Lei, H.; Wang, H.-Z. *J. Mater. Chem.* **2000**, *10*, 2698-2703.
